# Effect of Farmers’ Awareness of Climate Change on Their Willingness to Adopt Low-Carbon Production: Based on the TAM-SOR Model

**DOI:** 10.3390/ijerph20010619

**Published:** 2022-12-29

**Authors:** Jiaxu Ling, Yongji Xue, Chenyujing Yang, Yuanyuan Zhang

**Affiliations:** School of Economics and Management, Beijing Forestry University, Beijing 100083, China

**Keywords:** low-carbon agriculture, family farms, farmers’ willingness, climate change awareness

## Abstract

The COVID-19 pandemic highlighted the intricate relationships between human health and the social-ecological system in an era of climate and global change. Widespread COVID-19 adversely affected farmers’ employment, production practices, and livelihood resilience. At the same time, climate change is a key issue limiting agricultural production worldwide. Emissions of greenhouse gases, such as carbon dioxide, are a major factor leading to global climate change. Greenhouse gas emissions from agricultural production are receiving increasing attention. Therefore, it is particularly important to develop low-carbon agriculture. Based on data from 920 family farms in Jiangsu province and Shaanxi province, this study constructs a structural equation model and empirically tests the relationship between the variables using the bootstrap method. The results show that: (1) climate change awareness did not directly stimulate farmers’ willingness to pursue low-carbon production; (2) climate change awareness has an impact on low-carbon production willingness through perceived ease of use and consequence awareness; and (3) anti-risk ability can effectively moderate the impact of climate change awareness on low-carbon production behavior in agriculture. The theoretical model framework proposed in this study provides a reference for research in the field of low-carbon agriculture and also provides some insights and suggestions for environmentalists and governments. In addition, policymakers should effectively raise the sense of responsibility of farmers to address climate change and promote low-carbon agricultural production to achieve healthy and sustainable agricultural development.

## 1. Introduction

The COVID-19 pandemic is one of the most significant threats to global health since the Second World War [1]. The outbreak of COVID-19 has exposed the vulnerability of the current ecosystem, highlighting our faults and neglect towards disasters deriving from climate change or pollution [2]. COVID-19 and the climate crisis are global and one of the most urgent problems facing the world at present [3,4,5,6]. COVID-19 poses a threat to the health of human society; correspondingly, climate change is subtly worsening human health, and its negative impact cannot be ignored [7]. The United Nations Intergovernmental Panel on Climate change (IPCC) estimates that climate change may increase the risk of hunger and malnutrition by 20% by 2050 [8]. The epidemic has provided us with new ideas and insights to reflect on and deal with climate change [9]. Climate change and associated natural disasters are reshaping agricultural production and development patterns, thereby disturbing the resilience and sustainability of agriculture [10].

Climate change will also affect agricultural production. The main reason for the change in climate conditions is the increase in carbon dioxide emissions [11,12]. Existing research shows that the global temperature may rise by 1.5 °C in the next 20 years [12,13]. Global warming has led to an increase in extreme weather, seen in the frequency of natural disasters such as droughts and floods, and ecological degradation [14,15]. Agriculture as a sector is highly sensitive to global climate change, and extreme weather changes will adversely affect agricultural production [16]. In particular, in the face of natural disasters caused by irregular rainfall and other factors, agriculture is affected to a greater extent than other sectors [17,18]. Obviously, climate change has become a key factor limiting agricultural production. Farmers’ livelihoods are severely affected by extreme weather conditions, which lead to the reduction in the yield of major cash crops [19] and affect farmland and other production resources [20,21]. With continuous population growth, the imbalance between arable land and population may cause serious food insecurity [22]. Rapid climate change will also have a significant impact on agricultural productivity and changes in cropping patterns [23]. Climate change has significant impacts on agriculture. In developing countries, the agricultural sector is the important and indivisible sector of a country and is the material basis of other sectors. This has prompted us to constantly search for new methods and measures to reduce the impact of climate change on agricultural production and improve the adaptive capacity of farmers [24].

Low-carbon agriculture plays an important role in promoting sustainable agricultural development and coping with global climate change. The impact of climate change on multiple sectors is becoming increasingly significant. The increase in carbon dioxide emissions has attracted interest in low-carbon agriculture. It is necessary to study how to achieve a significant reduction in greenhouse gas emissions in the agricultural sector and reduce the impact on economic development [25]. Low-carbon agriculture can be defined as an agricultural system that can effectively produce food and raw materials, etc., reduce agricultural energy input and greenhouse gas emissions, and respect healthy and sustainable development [26]. Existing research shows that the carbon emissions of agriculture are second only to those of fossil fuel combustion [27,28]. This phenomenon is mainly caused by the heavy use of pesticides and fertilizers. Taking relevant actions to achieve the sustainable development goals has become a common demand throughout the contemporary world [29]. Low-carbon agriculture is extremely important for any country in the world [30]. Developing low-carbon agriculture is a way to alleviate pressure on ecological resources in rural areas and realize rural revitalization strategies [31]. The implementation of low-carbon agricultural technologies plays an important role in promoting farmers’ low-carbon behavior [32]. Low-carbon agriculture not only plays an important role in mitigating climate change, but can also bring about other developments such as green finance [33]. Planning to mitigate and adapt to the future impacts of climate change is complex. Technologies and measures for mitigating climate change need to be formulated according to specific conditions to minimize the adverse effects of climate change [34]. Low-carbon agriculture can balance the improvement of production efficiency and the coordination of economic and ecological benefits. It is a new path to achieving the healthy development of agriculture.

To explore farmers’ attitudes and intentions to adopt low-carbon production, in this study, we take stimulus–organism–response (SOR) as the theoretical framework, combine Technology Acceptance Model (TAM) theory in the field of farmers’ behavior research, and take farmers as the research subjects (farmers with family farms) to construct the influences of farmers’ awareness of climate change on their willingness to adopt low-carbon production. The purpose of this study is dual. First, we explore the effects of farmers’ willingness and behaviors on low-carbon production from the perspective of technology acceptance, and this work provides theoretical support and a reference for promoting the healthy, sustainable, and stable development of China’s low-carbon production. Second, this work provides empirical evidence and insights for agricultural managers, helping them to understand the ways in which farmers’ awareness of climate change affect their intentions to adopt low-carbon production.

## 2. Materials and Methods

### 2.1. Theoretical Analysis and Research Assumptions

Since the 1990s, and especially in the 21st century, many scholars have conducted research on farmers’ perception of climate change. These studies are mainly aimed at the farmers’ perception of climate change in different regions, their awareness of the causes and impacts of climate change, and the perceived characteristics of climate change [35,36]. Climate change awareness refers to the ability of individuals to recognize the nature of climate change problems, that is, awareness of temperature rises, sea level rises, extreme weather, and global warming [37]. The awareness of climate change among individuals helps to reduce different environmental problems [38,39]. In recent years, a growing number of scholars have realized that farmers are often rational in specific climate change risk scenarios. Farmers will take adaptive actions in combination with their own capabilities while pursuing maximum benefits [40,41]. Most farmers realize that the extreme weather caused by climate change has caused crop yield reduction, which has greatly hindered their production and livelihoods, and increased the uncertainty of agricultural input and output [42]. After realizing the impact of climate change on agricultural production, farmers tend to take measures to reduce the negative impact and must have expectations for the results of individual production behavior [43].

Consequence awareness refers to the individual’s perception of the possible positive or negative impacts of their own behavior [44]. In other words, if a person is aware of problems caused by certain behaviors, that awareness is followed by consideration of his or her own contribution to those problems and whether he or she can help to solve them [45,46]. Specifically, when individuals are aware that climate change will have an impact on agricultural production and that low-carbon production may have a positive impact on the environment, they tend to implement this behavior. This shows that individual consequence awareness is an important antecedent of low-carbon production willingness [47,48]. The analysis of climate change awareness and consequence awareness shows that no matter what happens before and after extreme weather, meta-cognition of climate change is the “fundamental factor” that positively affects the responsibility of mitigating climate change [49]. Farmers’ consequence awareness of low-carbon production behavior is the key factor for their willingness or unwillingness to take action [50,51].

Based on this, the following hypotheses are made in this study:

**Hypothesis** **1** **(H1):** *Climate change awareness has a positive and significant impact on consequence awareness;*

**Hypothesis** **2** **(H2):** *Climate change awareness has a positive and significant impact on low-carbon production willingness;*

**Hypothesis** **3** **(H3):** *Climate change awareness has a positive and significant impact on low-carbon production behavior;*

**Hypothesis** **4** **(H4):** *Consequence awareness has a positive and significant impact on low-carbon production willingness.*

The stimulus–organism–response model (SOR) is composed of three components: stimulus, organism, and behavioral response. The stimulus represents the external environmental factors, the organism as a mediating variable indicates the degree of perception of the individual, and the response represents the subject’s attitude or behavior [52,53,54]. The acceptance of low-carbon production technologies by agricultural production subjects is a key aspect of low-carbon agricultural production. Generally speaking, much of the literature on technology acceptance is provided by the Technology Acceptance Model (TAM), which is the causal model originally proposed by Davis [55,56]. The model is used to understand why individuals accept or reject a particular technology. Based on the rational relationships between belief, attitude, intention, and behavior, the model determines two key aspects of technology acceptance: perceived usefulness and perceived ease of use.

Perceived ease of use refers to the extent to which potential adopters believe that adopting a particular technology requires effort. Farmers need to invest significant energy and time to understand and learn new technologies. Potential adopters will first evaluate whether the new technology is easy to use through needs judgment, personal perception, and values. Perceived usefulness refers to the extent to which farmers believe that the use of specific new technologies will increase the effectiveness of their work and lead to increased returns. Generally speaking, farmers will judge and measure the internal and external costs and benefits of adopting agricultural production technology. This is one of the important factors affecting the willingness to adopt new technologies. In the TAM model, people’s beliefs about the usefulness and ease of use of technology will affect their intentions and end-use of technology. The TAM is often used in agricultural research to explore differences in technology adoption among farmers in developing countries [57,58]. At the same time, perceived usefulness and ease of use may also be influenced by external factors, such as personal cognitive changes resulting from training. Specifically, in the field of agricultural production, farmers’ climate change awareness affects their perceptions of the usefulness and ease of use of low-carbon production technologies [59]. The ideological tendency or motivation of farmers before implementing low-carbon behavior is low-carbon production willingness. Low-carbon production willingness is also the most direct antecedent factor of actual behavior. The stronger the farmers’ willingness for low-carbon production, the more likely they are to implement low-carbon production practices [60].

Based on this, the following hypotheses are made in this study:

**Hypothesis** **5** **(H5):** *Climate change awareness has a positive and significant impact on perceived ease of use;*

**Hypothesis** **6** **(H6):** *Climate change awareness has a positive and significant impact on perceived usefulness;*

**Hypothesis** **7** **(H7):** *Perceived ease of use has a positive and significant impact on low-carbon production willingness;*

**Hypothesis** **8** **(H8):** *Perceived usefulness has a positive and significant effect on low-carbon production willingness;*

**Hypothesis** **9** **(H9):** *Low-carbon production willingness has a positive and significant impact on low-carbon production behavior;*

**Hypothesis** **10** **(H10):** *Perceived ease of use has a positive and significant effect on perceived usefulness;*

**Hypothesis** **11** **(H11):** *Perceived usefulness has a positive and significant effect on consequence awareness.*

**Figure 1:**
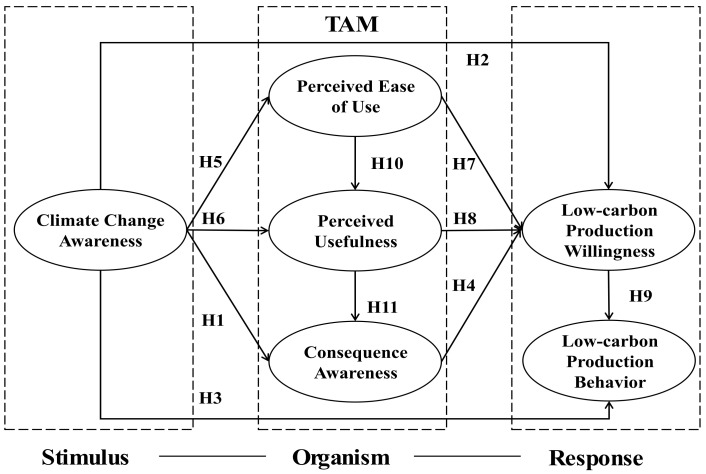
Theoretical model diagram.

Therefore, we obtain the theoretical model diagram as that in Figure 1. Overall, the proposed model is shown in Figure 1. Based on the SOR framework, we integrate the TAM to explore the effects of farmers’ awareness of climate change on their willingness to adopt low-carbon production. For this model figure, there are two points to be explained. Firstly, SOR theory emphasizes the effect of an external environmental stimulus on individual psychology. If the TAM is used without intuitive feelings (stimulus), there may be relatively large errors. On the contrary, if farmers are given external stimuli, it may be more rigorous and accurate to examine their willingness to adopt low-carbon production on the basis of awareness of climate change, which is exactly what the SOR model satisfies. Secondly, a single TAM is difficult to apply. Farmers have various perceptions and behaviors in the face of climate change, and there is even a link between these perceptions and behaviors. At the same time, the context of ‘organism’ in the SOR model is more extensive, and embedding the TAM may effectively improve the scientific research. Specifically, the awareness of climate change as an external stimulus arouses positive or negative emotional changes in farmers’ minds, thereby affecting their utility perception, and ultimately forming farmers’ adoption intention, which is the main research idea of this paper.

### 2.2. Data Sources

**Figure 2:**
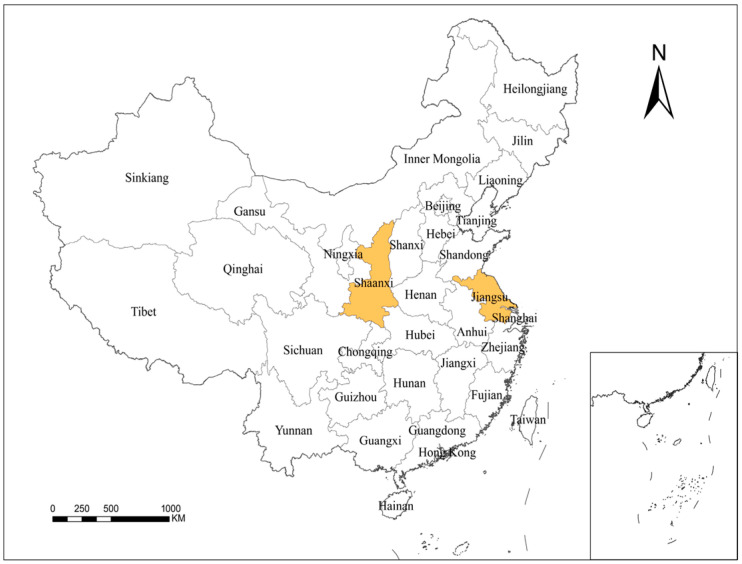
The location of the research area.

This study is based on family farms in Jiangsu province and Shaanxi province (Figure 2). The main reason for this choice is that the development of family farms in Jiangsu province and Shaanxi province has a promising future. Jiangsu province is a major agricultural province and an important major grain-producing area in China. In recent years, family farms in Jiangsu province have developed rapidly under the policy of cultivating new agricultural business entities [61]. Shaanxi province has continuously increased its efforts to promote the cultivation projects of new business entities, whose scale continues to grow [62]. In this study, quantitative analysis and empirical model hypothesis testing were used to analyze the relevant situation of family farm operators in terms of low-carbon production. The relevant data required were collected through an online questionnaire. The questionnaire used in this study consisted of two parts. The first part investigated the basic information of farmers, such as their age, education, years of family farm operation, and whether the person in charge of the family farm has farming experience. The second part investigated issues related to farmers’ climate change awareness (CCA), perceived ease of use (PEU), perceived usefulness (PU), consequence awareness (CA), anti-risk ability (AR), low-carbon production willingness (LW), and low-carbon production behavior (LB). This study uses the mature scale from international and national studies with a five-point Likert scale, making certain modifications in relation to the actual situation in China. We used simple sentences that farmers can understand to explain specific concepts.

The respondents were randomly selected farmers from Jiangsu Province and Shanxi Province in China. From January 2022 to February 2022, 1032 online questionnaires were collected. In all important quiz items, the required answer options were set to avoid omissions. Options such as “skip questions” were properly and reasonably set to ensure the effectiveness of the survey. Questionnaires with incomplete information or inaccurate answers were excluded; thus, 920 valid questionnaires were ultimately collected. There were 628 questionnaires from Jiangsu province and 292 questionnaires from Shaanxi province. The effectiveness rate of the questionnaire was 89.15%.

The basic information of the farmers is shown in Table 1. In terms of age distribution, the majority were aged 41–60, accounting for 74.13% of the total sample. In terms of educational level, there were more high-school and secondary school respondents, accounting for 46.63% of the total sample, which is largely in line with the current educational level in rural areas. From the perspective of family farm operating lifetime, 34.89% of family farms had been in operation for 5–7 years. Most family farm leaders had farming experience, accounting for 91.96%, and only 74 family farm leaders had no farming experience. In summary, the sample distribution is roughly representative of the actual situation, and the structure is reasonable and representative.

### 2.3. Study Results

The average and standard deviation were calculated for each observed variable of farmers’ combined attitudes using SPSS 26.0 software (IBM, New York, NY, USA) (Table 2). It was found that perceived ease of use (PEU), perceived usefulness (PU), consequence awareness (CA), and risk resistance (AR) all have mean values of 3 or greater. This shows that farmers think that climate change will have an impact on agricultural production. Global warming has led to increased instability in crop production, and the yield and quality of grain are also affected and impacted. Low-carbon production can help to improve this situation, slow down the impact of climate change on agriculture, enhance the adaptability of agriculture, and promote the sustainable and healthy development of agriculture. According to the various items measuring low-carbon production willingness (LW) and low-carbon production behavior (LB), it is clear that farmers have a strong willingness for low-carbon production, but their actual actions are relatively limited.

## 3. Results

This study aims to explore the impact of various variables of farmers’ climate change awareness on their low-carbon production willingness and behaviors. The advantage of the structural equation model (SEM) is that it can take into account and deal with multiple dependent variables at the same time and can effectively explain latent variables such as attitudes and behaviors. Therefore, this study used the SEM to analyze the research problem. The analysis steps include four stages: a reliability and validity test, an SEM test, mediation effect analysis, and moderating effect analysis.

### 3.1. Reliability and Validity Test

Reliability is a method to measure content consistency and reliability. Cronbach’s α coefficient is generally selected as the measurement standard. It is generally believed that a value between 0.7–0.8 means good reliability, while more than 0.8 means very good. Table 3 shows that the overall questionnaire’s Cronbach’s α coefficient was 0.954, and the Cronbach’s α of each item was above 0.7. The results show that the test had a high degree of stability and reliability.

The Kaiser–Meyer–Olkin (KMO) test and Bartlett’s sphericity test were used to determine whether the questionnaire was reasonably valid and met the expectations of the study. It is generally believed that, if the KMO test value is > 0.6 and *p* < 0.01 or *p* < 0.05, the results are significant, and a factor analysis can be carried out. As shown in Table 3, the KMO test value is 0.936, and the Bartlett’s spherical test results show that the test was significant. The factor analysis was effective, and the degree was appropriate.

The criterion for convergent validity in this study is that the square root of the AVE of the latent variable is greater than the correlation coefficient under the other constructs. It can be seen that there was a strong correlation among the items in the variable group, which indicates that the convergence of the questionnaire was ideal (Table 4).

### 3.2. Application of Structural Equation Model

Since the reliability and validity tests were all good, a path analysis was conducted for the model. The final fit results are shown in Table 5. By observing the fitting index, we can determine that the model proposed in this study is good, which is consistent with the actual survey data (Table 6). From the significance level of each path fitting (Table 6), all the other paths reached the significance level, except for “CCA--->LW”, with a significant value of 0.110, “CCA--->LB”, with a significant value of 0.544, and “CCA--->PU, with a significant value of 0.207. In terms of the theoretical analysis, there should be a positive correlation between climate change awareness and low-carbon production. Therefore, the model needs to be modified according to the actual situation of farmers.

### 3.3. Intermediary Effect Analysis

At present, there are three main types of intermediary utility tests: the causal step test, the coefficient multiplication method (Sobel test, Bootstrap test), and the difference coefficient test [63]. The Sobel test and the coefficient of variation test are not applicable in models with multiple mediating variables, and their formulas are complex and suffer from a high Type I error rate. Combined with the analysis of the actual situation of this study and referring to the multiple intermediary effect analysis program proposed by Fang [64], the bootstrap method of AMOS 24.0 was used to test the specific intermediary effect. This study set the bootstrap to 5000 runs. Climate change awareness was used as an independent variable to analyze the intermediary effect of the specific path. Four paths were defined: M1a (CCA--->PEU--->LW), M2b (CCA--->PEU--->PU--->LW), M3c (CCA--->PEU--->PU--->CA--->LW), and M4d (CCA--->CA--->LW). The running results are shown in Table 7. The specific mediating effect M1a on climate change awareness through perceived ease of use to low-carbon production willingness is 0.127. Bias-corrected 95% CI = [0.083, 0.169], excluding 0, meaning that the intermediary path was established. In the same way, the specific mediating effect M2b of climate change awareness through perceived ease of use and perceived usefulness to low-carbon production willingness was 0.097. Bias-corrected 95% CI = [0.063, 0.131], meaning that the path has statistical significance. The specific mediating effect M3c of climate change awareness through perceived ease of use, perceived usefulness, and consequence awareness to low-carbon production willingness was 0.035. Bias-corrected 95% CI = [0.021, 0.050], meaning that the path has statistical significance. The specific mediating effect M4d of climate change awareness through consequence awareness to low-carbon production willingness was 0.030. Bias-corrected 95% CI = [0.021, 0.055], meaning that the path has statistical significance.

### 3.4. Mediated Moderator

This study considers the introduction of moderating variables in order to address the unstable effect of farmers’ climate change awareness on low-carbon production willingness. From the above test results, it can be seen that the direct impact of climate change awareness on low-carbon production willingness is not significant, but the intermediary impacts of perceived usefulness and consequence awareness are significant. Therefore, SPSS 26.0 and Model 58 were used in the process model diagram compiled to analyze the mediated moderator effect. Many scholars have put forward the important role of anti-risk ability (AR) in the decision-making process of farmers. Different farmers have different attitudes towards risks, which is manifested in the negative impact of low risk avoidance and adaptive strategies [65]. The stronger the farmers’ risk awareness, the more inclined they are to manage climate risks [66]. In the face of the impact of natural disasters, anti-risk ability has a significant positive moderating effect on farmers’ decision making [67].

**Figure 3:**
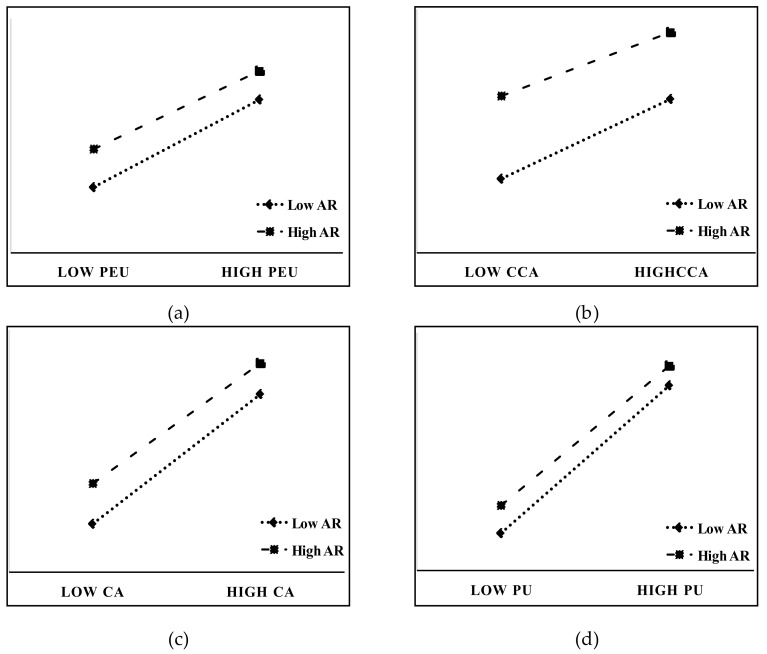
Mediated moderator effect. (**a**) Regulating Action Diagram (PEU-LW); (**b**) Regulating Action Diagram (CCA-CA); (**c**) Regulating Action Diagram (CA-LW); (**d**) Regulating Action Diagram (PU-LW).

With perceived ease of use (PEU) as the mediating variable and anti-risk ability (AR) as the moderating variable, a path test was carried out (Table 8, Figure 3a). The results show that the regulatory effect of AR on the CCA--->PEU pathway was not significant (interactive items se = 0.011, t = −0.617, *p* = 0.537), but the regulation of the PEU--->LW pathway was significant (interactive item se = 0.022, t = −2.249, *p* < 0.050).

With consequence awareness (CA) as the mediating variable and anti-risk ability (AR) as the moderating variable, a path test was carried out (Table 9, Figure 3b,c). The results show that the regulatory effect of anti-risk ability (AR) on the CCA--->CA pathway was significant (interactive items se = 0.017, t = −4.681, *p* < 0.001). Furthermore, the regulation of the CA--->LW pathway was significant (interactive items se = 0.024, t = −1.990, *p* < 0.050).

Model 87 was used in the process model diagram to analyze the moderating effects on specific mediation paths (CCA--->PEU--->PU--->LW). With perceived ease of use (PEU) as M1, perceived usefulness (PU) as M2, and anti-risk ability (AR) as the moderating variable, a path test was carried out (Table 10, Figure 3d). The results show that the regulatory effect of anti-risk ability (AR) on the PU--->LW pathway was significant (interactive items se = 0.017, t = −2.471, *p* < 0.05).

## 4. Conclusions

This paper explores the formation mechanism of farmers’ low-carbon production willingness under the background of climate change by building a theoretical model of the relationship between climate change awareness (stimulus), perceived ease of use, perceived usefulness, consequence awareness (organism), willingness to adopt low-carbon agricultural technologies, and behavior (response). Furthermore, the moderating role of farmers’ anti-risk ability on low-carbon agricultural production was analyzed. The results of the empirical analysis strongly support the overall research model proposed in this study.

### 4.1. Climate Change Awareness Did Not Directly Stimulate Farmers’ Willingness to Adopt Low-Carbon Production

This study confirms that climate change awareness did not directly affect farmers’ low-carbon production willingness or behavior. Our results are similar to previous studies [68]. In other words, even though farmers are aware of climate change effects, they might not engage in adaptation measures. Climate change is a global issue of universal concern to the international community. In recent years, extreme weather events such as global heat, drought, and floods, etc., have occurred frequently. These have had a serious impact on the development of various countries. Our research finds that climate change does not directly promote the low-carbon transformation in technology and production. At least in the agricultural field, it does not directly bring about technological change and production transformation.

### 4.2. Climate Change Awareness Has an Impact on Low-Carbon Production Willingness through Perceived Ease of Use and Consequence Awareness

Firstly, the perceived ease of use of low-carbon agricultural technology and the consequence awareness of low-carbon production can significantly and positively influence farmers’ willingness to adopt low-carbon production. Our results are similar to Li et al. [69]. On the one hand, perceived ease of use can promote the willingness of low-carbon agricultural production. The more farmers perceive that low-carbon production technology is convenient to use, the higher their willingness to adopt low-carbon production. On the other hand, the role of consequence awareness in the adoption of low-carbon technologies has also been confirmed. When farmers perceive that the benefits brought by low-carbon agricultural production are better, their willingness to participate in low-carbon agricultural production is higher.

Secondly, the acceptance of low-carbon agricultural technology can mediate the impact of the climate stimulus on the willingness to adopt low-carbon agricultural production. That is, climate change can improve farmers’ perception of low-carbon technologies and then stimulate their willingness to use low-carbon technologies in agriculture. Specifically, the perceived usefulness of technology and the perceived ease of use of technology can play an intermediary role together to provide a path for the transmission of climate change.

### 4.3. Anti-Risk Ability Can Effectively Moderate the Impact of Climate Change Awareness on Low-Carbon Production Behavior in Agriculture

For farmers with a strong anti-risk ability, climate change awareness has a strong impact on low-carbon willingness. Our results are consistent with Tesfaye et al. [67]. Specifically, farmers with a strong anti-risk ability are more comfortable with crises and usually have the capital and capacity to cope with external factors. Farmers with a low risk resistance are more sensitive to the risks of low-carbon agriculture technology adoption. From the empirical analysis, anti-risk ability can positively moderate the pathways of “climate change awareness–consequence awareness”, “consequence awareness–behavior willingness”, “perceived usefulness–behavior willingness”, and “perceived ease of use–behavior willingness”. The stronger the risk-taking ability of farmers, the stronger their willingness to adopt environmentally friendly technologies. Conversely, farmers with a weak anti-risk ability have stricter requirements in terms of ease of use and the usefulness of low-carbon production.

## 5. Contribution and Limitations

The above research results provide some contributions to the promotion and technological development of low-carbon agriculture, as well as useful insights for agricultural managers.

### 5.1. Theoretical Contribution

The important theoretical contribution of this study is to prove the applicability of the integration of the SOR framework and the TAM in the field of low-carbon agricultural production. Through a review of related research, it was found that the applicability of the SOR framework and the TAM in the field of low-carbon agricultural production has not been effectively confirmed. The relationship between the two structures has also not been empirically resolved. This study reveals changes in farmers’ perception of low-carbon production stimulated by climate change and how this perception affects farmers’ behavior in terms of low-carbon production. In fact, the transformation of agricultural production is influenced by many external stimuli. The SOR framework can be further applied to research on changes in agricultural methods and technological transformations of agricultural production due to stimulating factors such as COVID-19, policy changes, and market fluctuations. Generally speaking, the SOR framework provides a reference and new ideas for research in the field of low-carbon agriculture and broadens the research boundary.

### 5.2. Application Value

This study provides some references for environmental workers and low-carbon agricultural technology developers. Our research shows that the indirect influence of climate change awareness on agricultural low-carbon production willingness and the role of climate change education in actual production. This can enhance the confidence of environmental workers in raising public awareness of climate change. Environmental workers can take various measures, such as knowledge lectures, to enhance the farmers’ climate policy awareness and raise the publicity of the relevant knowledge of climate policy [70]. Positive promotional content will also inspire farmers to establish a stable, positive emotional connection to the concept of low-carbon production. It is conducive to promoting low-carbon travel among the public. At the same time, we found that perceived ease of use and consequence awareness had a significant effect on willingness and behavior in terms of low-carbon agriculture production. This suggests that developers of low-carbon agricultural technologies need to pay more attention to farmers’ experience in their work in order to promote low-carbon production willingness. Developers of low-carbon agricultural technologies can also promote farmers’ recognition and willingness to adopt low-carbon technologies by introducing the use of low-carbon technologies to farmers in detail.

In addition, we found that anti-risk ability is useful in addressing the impact between climate change and low-carbon agricultural production. This provides some reference for government workers, especially in China. In recent years, the Chinese government has been attempting to increase the share of family farms and cooperatives in agricultural production, hoping to bring about the adoption of low-carbon technologies through scale production. In fact, our research shows the feasibility of improving anti-risk ability to promote low-carbon production. Compared with small farmers, it is relatively easy for family farms to obtain low-carbon agriculture production resources [71]. Scale production usually means a strong ability to resist risks. For government managers, this can be considered in the future to improve farmers’ anti-risk ability by means of scale, thereby promoting the transformation to low-carbon agricultural production.

### 5.3. Limitations and Future Studies

Although this study has made contributions in theory and practice, it still presents limitations. The first disadvantage stems from the cross-sectional nature of the data studied. Due to the limitation of time and resources, we used cross-sectional data from only two provinces in China. This conclusion still has room for optimization. At the same time, due to the regional limitations of the data, the differences of some variables are not reflected in the model. For example, farmers in different regions may have different perceptions of the usefulness of low-carbon agriculture production. Often, in more economically developed areas, the threshold for the use of low-carbon technologies is relatively lower. In future research, we can consider using panel data for analysis to broaden the applicability of the model. The second weakness lies in the inadequate exploration of the relationship between low-carbon production willingness and low-carbon production behavior. The transformation mechanism from willingness to behavior should also be the focus of future research.

## Figures and Tables

**Table 1:** Sample basic information.

Variable	Classification	Frequency	Percentage
Age	Under 30	16	1.74%
	31–40	203	22.07%
	41–50	386	41.96%
	51–60	296	32.17%
	Above 60	19	2.06%
Education	Primary school and below	7	0.76%
	Junior high school	235	25.54%
	High school/Technical secondary school	429	46.63%
	Junior college/Undergraduate	242	26.30%
	Master degree and above	7	0.77%
Years of family farm operation	1–2	89	9.67%
	3–4	168	18.26%
	5–7	321	34.89%
	8–10	235	25.54%
	Above 11	107	11.64%
Farming experience of family farm leaders	Yes	846	91.96%
	No	74	8.04%

**Table 2:** Descriptive statistical analysis of the questionnaire.

Variable	Variable Measure	Code	Average	Standard Deviation
Climate change awareness	Do you think climate change has an impact on agriculture?	CCA1	2.900	1.209
	Do you think climate change has an impact on the farm’s economic development?	CCA2	2.820	1.169
Perceived ease of use	I am familiar with the relevant skills and means of low-carbon management.	PEU1	3.350	1.584
	I think it is not difficult to adopt a low-carbon management mode in land farming.	PEU2	3.340	1.526
	I am familiar with the relevant national and local policies on low-carbon agricultural production.	PEU3	3.500	1.487
Perceived usefulness	I think, compared with the traditional agricultural management, the low-carbon agricultural products obtained from low-carbon management can bring a higher economic income.	PU1	3.530	1.493
	I think low-carbon production can provide high-quality and safe agricultural products (food, oil, vegetables, etc.).	PU2	3.570	1.477
	I think low-carbon production can improve the fertility of cultivated land and ensure the long-term high output capacity of cultivated land.	PU3	3.790	1.436
	I think low-carbon production can effectively reduce greenhouse gas emissions.	PU4	3.810	1.429
Consequence awareness	I think low-carbon agricultural production can mitigate the disaster of climate change.	CA1	3.800	1.44
	I think that conducting low-carbon agricultural production can prevent agricultural pollution.	CA2	4.070	1.314
	Our survival and development need to be based on the protection of the natural environment.	CA3	4.100	1.323
	I think the low-carbon agricultural production can effectively protect the environment.	CA4	4.170	1.277
Anti-risk ability	I can afford the capital cost of low-carbon business (such as buying green pesticides, straw returning equipment, etc.).	AR1	4.120	1.318
	I am able to withstand economic risks in low-carbon operation (such as sales risk of low-carbon agricultural products).	AR2	3.970	1.423
	If I try my best, I can solve most of the problems I encounter.	AR3	4.010	1.413
Low-carbon production willingness	I am willing to reduce the use of chemical fertilizers (or use organic fertilizer).	LW1	3.860	1.544
	I am willing to reduce pesticide use (or use green pesticides).	LW2	4.020	1.422
	I am willing to reduce the use of mulch.	LW3	3.280	1.536
	I am willing to adopt a new comprehensive agricultural mode, such as agricultural energy-saving production mode, waste recycling mode.	LW4	3.310	1.569
Low-carbon production behavior	I reduced the use of fertilizer (or used organic fertilizer).	LB1	2.650	1.692
	I reduced pesticide use (or used green pesticides).	LB2	4.000	1.751
	I returned farmland to forest or returned farmland to grass.	LB3	3.780	2.245

**Table 3:** Reliability and validity test of questionnaire.

Variable	Variable Measure	Factor Loading	AVE	C.R.	α
CCA	CCA1	0.844	0.918	0.914	0.908
	CCA2	0.987			
PEU	PEU1	0.922	0.895	0.924	0.922
	PEU2	0.882			
	PEU3	0.881			
PU	PU1	0.877	0.930	0.962	0.961
	PU2	0.940			
	PU3	0.962			
	PU4	0.938			
CA	CA1	0.977	0.945	0.971	0.971
	CA2	0.916			
	CA3	0.961			
	CA4	0.925			
LW	LW1	0.936	0.926	0.960	0.938
	LW2	0.951			
	LW3	0.894			
	LW4	0.923			
LB	LB1	0.464	0.734	0.768	0.781
	LB2	0.836			
	LB3	0.838			
Overall reliability of the questionnaire (Cronbach’s α) = 0.954					
KMO = 0.936; Bartlett = 20750.81; df = 190; Sig.= 0.000					

**Table 4:** Differential validity test.

	CCA	PEU	PU	CA	LW	LB
CCA	**0.918**	-	-	-	-	-
PEU	0.205	**0.895**	-	-	-	-
PU	0.186	0.762	**0.865**	-	-	-
CA	0.247	0.702	0.718	**0.930**	-	-
LW	0.203	0.561	0.733	0.665	**0.926**	
LB	0.155	0.577	0.645	0.525	0.681	**0.734**

Note: Bold values are the square root of the AVE.

**Table 5:** Pathway hypothesis test results.

	Std.	S.E.	C.R.	*p*
PEU<---CCA	0.208	0.047	6.047	***
PU<---CCA	0.030	0.030	1.261	0.207
PU<---PEU	0.762	0.027	25.868	***
CA<---CCA	0.119	0.031	4.901	***
CA<---PU	0.702	0.027	25.647	***
LW<---PEU	0.107	0.037	2.703	**
LW<---PU	0.602	0.050	12.526	***
LW<---CA	0.293	0.036	8.464	***
LW<---CCA	0.038	0.031	1.600	0.110
LB<---LW	0.687	0.032	22.100	***
LB<---CCA	0.017	0.037	0.607	0.544

Note: *** *p* < 0.001, ** *p* < 0.01.

**Table 6:** Structural equation model fitting results.

Inspection Index	CMIN/DF	GFI	AGFI	NFI	IFI	CFI	RMSEA
Model Estimates	4.973	0.925	0.900	0.962	0.970	0.970	0.066
Criterion for Judgement	1–5	>0.800	>0.800	>0.800	>0.800	>0.800	<0.080

**Table 7:** Specific intermediary path test.

Specific Mediation Path	Estimate	*p*	Lower	Upper	Established
M1a (CCA--->PEU--->LW)	0.127	0.000	0.083	0.169	Yes
M2b (CCA--->PEU--->PU--->LW)	0.097	0.000	0.063	0.131	Yes
M3c (CCA--->PEU--->PU--->CA--->LW)	0.035	0.000	0.021	0.050	Yes
M4d (CCA--->CA--->LW)	0.030	0.001	0.021	0.055	Yes

**Table 8:** Test of moderating effect with PEU as mediating variable.

	coeff	se	t	*p*	coeff	se	t	*p*
constant	0.366	0.122	3.003	**	1.554	0.218	7.135	***
CCA	0.050	0.042	1.191	0.2341	0.087	0.033	2.647	**
AR	0.874	0.034	25.494	***	0.356	0.093	5.094	***
PEU					0.468	0.092	3.846	***
CCA × AR	−0.007	0.011	−0.617	0.537				
PEU × AR					−0.050	0.022	−2.249	*
R-sq	0.8137				0.305			
F	1333.716				100.208			

Note: *** *p* < 0.001, ** *p* < 0.01, * *p* < 0.05.

**Table 9:** Test of moderating effect with CA as mediating variable.

	coeff	se	t	*p*	coeff	se	t	*p*
constant	1.123	0.186	6.033	***	0.621	0.235	2.640	**
CCA	0.409	0.065	6.3427	***	0.019	0.030	0.629	0.530
CA					0.670	0.057	11.727	***
AR	0.767	0.052	14.620	***	0.368	0.115	3.205	**
CCA × AR	−0.081	0.017	−4.681	***				
CA × AR					−0.048	0.024	−1.990	*
R-sq	0.459				0.448			
F	258.951				185.556			

Note: *** *p* < 0.001, ** *p* < 0.01, * *p* < 0.05.

**Table 10:** Test of moderating effect.

	coeff	se	t	*p*
constant	0.866	0.188	4.605	***
CCA	0.053	0.028	1.904	0.057
PEU	−0.063	0.055	−1.153	0.249
PU	0.777	0.058	13.398	***
AR	0.249	0.082	3.030	**
PU × AR	−0.042	0.017	−2.471	*
R-sq	0.508			
F	188.866			

Note: *** *p* < 0.001, ** *p* < 0.01, * *p* < 0.05.

## References

[1] Raza T., Shehzad M., Abbas M., Eash N.S., Jatav H.S., Sillanpaa M., Flynn T. (2022). Impact Assessment of COVID-19 Global Pandemic on Water, Environment, and Humans. Environ. Adv..

[2] Marazziti D., Cianconi P., Mucci F., Foresi L., Chiarantini I., Della Vecchia A. (2021). Climate change, environment pollution, COVID-19 pandemic and mental health. Sci. Total Environ..

[3] Rosenbloom D., Markard J. (2020). A covid-19 recovery for climate. Science.

[4] Wang Y., Eliot M.N., Koutrakis P., Gryparis A., Schwartz J.D., Coull B.A., Mittleman M.A., Milberg W.P., Lipsitz L.A., Wellenius G.A. (2014). Ambient air pollution and depressive symptoms in older adults: Results from the MOBILIZE Boston study. Environ. Health Perspect..

[5] Conticini E., Frediani B., Caro D. (2020). Can atmospheric pollution be considered a co-factor in extremely high level of SARS-CoV-2 lethality in northern Italy. Environ. Pollut..

[6] Ahmed W., Hoffmann L.M., Al-Hasani T., Santos R.M. (2022). Impact of the COVID-19 Pandemic on the 2020 Diurnal Temperature Range (DTR) in the Contiguous USA. Atmosphere.

[7] Zang S.M., Benjenk I., Breakey S., Pusey-Reid E., Nicholas P.K. (2021). The intersection of climate change with the era of COVID-19. Public Health Nurs..

[8] Howarth C., Viner D., Dessai S., Rapley C., Jones A. (2017). Enhancing the Contribution and Role of Practitioner Knowledge in the Intergovernmental Panel on Climate Change (IPCC) Working Group (WG) II Process: Insights from UK Workshops. Clim. Serv..

[9] Manzanedo R.D., Manning P. (2020). COVID-19: Lessons for the climate change emergency. Sci. Total Environ..

[10] Song Y., Zhang B., Wang J., Kwek K. (2022). The impact of climate change on China’s agricultural green total factor productivity. Technol. Forecast. Soc. Chang..

[11] Hoegh-Guldberg O., Jacob D., Taylor M., Bolaños T.G., Bindi M., Brown S., Camilloni I.A., Diedhiou A., Djalante R., Ebi K. (2019). The human imperative of stabilizing global climate change at 1.5 C. Science.

[12] Zeng X., Guo S., Deng X., Zhou W., Xu D. (2021). Livelihood risk and adaptation strategies of farmers in earthquake hazard threatened areas: Evidence from Sichuan province, China. Int. J. Disaster Risk Reduct..

[13] Ma J., Zhou W., Guo S., Deng X., Song J., Xu D. (2022). Effects of Conformity Tendencies on Farmers’ Willingness to Take Measures to Respond to Climate Change: Evidence from Sichuan Province, China. Int. J. Environ. Res. Public Health..

[14] Budhathoki N.K., Paton D., Lassa J.A., Zander K.K. (2020). Assessing farmers’ preparedness to cope with the impacts of multiple climate change-related hazards in the Terai lowlands of Nepal. Int. J. Disaster Risk Reduct..

[15] Guo J., Chen J. (2022). The Impact of Heavy Rainfall Variability on Fertilizer Application Rates: Evidence from Maize Farmers in China. Int. J. Environ. Res. Public Health.

[16] Minh H.V.T., Kumar P., Van Ty T., Duy D.V., Han T.G., Lavane K., Avtar R. (2022). Understanding Dry and Wet Conditions in the Vietnamese Mekong Delta Using Multiple Drought Indices: A Case Study in Ca Mau Province. Hydrology.

[17] Ahsan F., Chandio A.A., Fang W. (2020). Climate change impacts on cereal crops production in Pakistan: Evidence from cointegration analysis. Int. J. Clim. Chang. Strateg. Manag..

[18] Ul-Haq Z., Mehmood U., Tariq S., Qayyum F., Azhar A., Nawaz H. (2022). Analyzing the role of meteorological parameters and CO_2_ emissions towards crop production: Empirical evidence from South Asian countries. Environ. Sci. Pollut. Res..

[19] Khan N., Ma J., Kassem H.S., Kazim R., Ray R.L., Ihtisham M., Zhang S. (2022). Rural Farmers’ Cognition and Climate Change Adaptation Impact on Cash Crop Productivity: Evidence from a Recent Study. Int. J. Environ. Res. Public Health.

[20] Chandio A.A., Jiang Y., Rehman A., Rauf A. (2020). Short and long-run impacts of climate change on agriculture: An empirical evidence from China. Int. J. Clim. Chang. Strateg. Manag..

[21] Ozdemir D. (2021). The Impact of Climate Change on Agricultural Productivity in Asian Countries: A heterogeneous panel data approach. Environ. Sci. Pollut. Res..

[22] Abbas S. (2022). Climate change and major crop production: Evidence from Pakistan. Environ. Sci. Pollut. Res..

[23] Kumar R., Gautam H.R. (2014). Climate change and its impact on agricultural productivity in India. J. Climatol. Weather Forecast..

[24] Chandio A.A., Nasereldin Y.A., Anh D.L.T., Tang Y., Sargani G.R., Zhang H. (2022). The impact of technological progress and climate change on food crop production: Evidence from Sichuan—China. Int. J. Environ. Res. Public Health.

[25] Lehtonen H., Huan-Niemi E., Niemi J. (2022). The transition of agriculture to low carbon pathways with regional distributive impacts. Environ. Innov. Soc. Transit..

[26] Piwowar A. (2019). Low carbon agriculture in Poland–theoretical and practical challenges. Pol. J. Environ. Stud..

[27] Liu M., Yang L. (2021). Spatial pattern of China’s agricultural carbon emission performance. Ecol. Indic..

[28] Aguilera E., Reyes-Palomo C., Díaz-Gaona C., Sanz-Cobena A., Smith P., García- Laureano R., Rodríguez-Est’evez V. (2021). Greenhouse gas emissions from Mediterranean agriculture: Evidence of unbalanced research efforts and knowledge gaps. Global Environ. Chang..

[29] Zhou H., Bhattarai R., Li Y., Si B., Dong X., Wang T., Yao Z. (2022). Towards sustainable coal industry: Turning coal bottom ash into wealth. Sci. Total Environ..

[30] Borychowski M., Grzelak A., Popławski Ł. (2022). What drives low-carbon agriculture? The experience of farms from the Wielkopolska region in Poland. Environ. Sci. Pollut. Res..

[31] Zhu J. (2022). Dynamic Evaluation of China’s Low-carbon Agriculture Development Level from the Perspective of Fuzzy Incentives and Punishments. For. Chem. Rev..

[32] Yang X., Zhou X., Deng X. (2022). Modeling farmers’ adoption of low-carbon agricultural technology in Jianghan Plain, China: An examination of the theory of planned behavior. Technol. Forecast. Soc. Chang..

[33] van Veelen B. (2021). Cash cows? Assembling low-carbon agriculture through green finance. Geoforum..

[34] Malhi G.S., Kaur M., Kaushik P. (2021). Impact of climate change on agriculture and its mitigation strategies: A review. Sustainability..

[35] Bryan E., Deressa T.T., Gbetibouo G.A., Ringler C. (2009). Adaptation to climate change in Ethiopia and South Africa: Options and constraints. Environ. Sci. Policy..

[36] Habiba U., Shaw R., Takeuchi Y. (2012). Farmer’s perception and adaptation practices to cope with drought: Perspectives from Northwestern Bangladesh. Int. J. Disaster Risk Reduction..

[37] Lawrance E., Thompson R., Fontana G., Jennings N. (2021). The Impact of Climate Change on Mental Health and Emotional Wellbeing: Current Evidence and Implications for Policy and Practice. https://www.imperial.ac.uk/grantham/publications/all-publications/the-impact-of-climate-change-on-mentalhealth-and-emotional-wellbeing-current-evidence-and-implications-for-policy-and-practice.php.

[38] Zhao Z., Xue Y., Xu Y., Ndongo N. (2022). The Influence of Environmental Values on Consumer Intentions to Participate in Agritourism—A Model to Extend TPB. J. Agric. Environ. Ethics.

[39] Fu L., Sun Z., Zha L., Liu F., He L., Sun X., Jing X. (2020). Environmental awareness and pro-environmental behavior within China’s road freight transportation industry: Moderating role of perceived policy effectiveness. J. Clean. Prod..

[40] Ma J., Zhou W., Guo S., Deng X., Song J., Xu D. (2022). Space-time perception and behavioral response of farmers to climate change: Evidence from Sichuan Province, China. Front. Ecol. Evol..

[41] Tiet T., Nguyen-Anh T. (2022). Farmers’ behaviors and attitudes toward climate change adaptation: Evidence from Vietnamese smallholder farmers. Environ. Dev. Sustain..

[42] Zamasiya B., Nyikahadzoi K., Mukamuri B.B. (2017). Factors influencing smallholder farmers’ behavioural intention towards adaptation to climate change in transitional climatic zones: A case study of Hwedza District in Zimbabwe. J. Environ. Manag..

[43] Van Winsen F., de Mey Y., Lauwers L., Van Passel S., Vancauteren M., Wauters E. (2016). Determinants of risk behaviour: Effects of perceived risks and risk attitude on farmer’s adoption of risk management strategies. J. Risk Res..

[44] Yazdanmehr A., Wang J. (2016). Employees’ information security policy compliance: A norm activation perspective. Decision Support Systems..

[45] Joanes T. (2019). Personal norms in a globalized world: Norm-activation processes and reduced clothing consumption. J. Clean. Prod..

[46] Zobeidi T., Yaghoubi J., Yazdanpanah M. (2022). Exploring the motivational roots of farmers’ adaptation to climate change-induced water stress through incentives or norms. Sci. Rep..

[47] Perera C.R., Kalantari H., Johnson L.W. (2022). Climate change beliefs, personal environmental norms and environmentally conscious behaviour intention. Sustainability..

[48] Hallaj Z., Sadighi H., Farhadian H., Bijani M. (2021). Human ecological analysis of farmers’ pro-environmental behaviour in the face of drought: Application of Norm Activation Theory. Water Environ. J..

[49] Wu W., Zheng J., Fang Q. (2020). How a typhoon event transforms public risk perception of climate change: A study in China. J. Clean. Prod..

[50] Barnes A.P., Toma L. (2012). A typology of dairy farmer perceptions towards climate change. Clim. Chang..

[51] Arbuckle J.G., Morton L.W., Hobbs J. (2013). Farmer beliefs and concerns about climate change and attitudes toward adaptation and mitigation: Evidence from Iowa. Clim. Chang..

[52] Luqman A., Cao X., Ali A., Masood A., Yu L. (2017). Empirical Investigation of Facebook Discontinues Usage Intentions Based on SOR Paradigm. Comput. Hum. Behav..

[53] Lee H.J., Yun Z.S. (2015). Consumers’ perceptions of organic food attributes and cognitive and affective attitudes as determinants of their purchase intentions toward organic food. Food Qual. Prefer..

[54] Wu Y.L., Li E.Y. (2018). Marketing mix, customer value, and customer loyalty in social commerce: A stimulus-organism-response perspective. Int. Res..

[55] Davis F.D. (1989). Perceived usefulness, perceived ease of use, and user acceptance of information technology. Manag. Inf. Syst..

[56] Davis F.D., Bagozzi R.P., Warshaw P.R. (1989). User Acceptance of Computer Technology: A Comparison of Two Theoretical Models. Manag. Sci..

[57] Khoza S., de Beer L.T., van Niekerk D., Nemakonde L. (2021). A gender-differentiated analysis of climate-smart agriculture adoption by smallholder farmers: Application of the extended technology acceptance model. Gend. Technol. Dev..

[58] Kabir K.H., Hassan F., Mukta M.Z.N., Roy D., Darr D., Leggette H., Ullah S.M.A. (2022). Application of the technology acceptance model to assess the use and preferences of ICTs among field-level extension officers in Bangladesh. Digit. Geogr. Soc..

[59] Le Dang H., Li E., Bruwer J., Nuberg I. (2014). Farmers’ perceptions of climate variability and barriers to adaptation: Lessons learned from an exploratory study in Vietnam. Mitig. Adapt. Strateg. Glob. Chang..

[60] Nguyen N., Drakou E.G. (2021). Farmers intention to adopt sustainable agriculture hinges on climate awareness: The case of Vietnamese coffee. J. Clean. Prod..

[61] Cao T.Y., Wang X.Q., Wei Z. (2020). Farm operation scale, agricultural technical training, and farm income in Jiangsu. Res. Agric. Mod..

[62] Bai F.J., Shen S.B., Huang P.Y., Bai M.M., Jiang Y.H., Yan R. (2022). An Analysis of the Path Selection of Leading Industries for sssRural Revitalization in Shaanxi Province After Poverty Alleviation. J. Xianyang Norm. Univ..

[63] Wen Z., Fang J., Xie J., Ouyang J. (2022). Methodological research on mediation effects in China’s mainland. Adv. Psychol. Sci..

[64] Fang J., Wen Z. (2022). Moderation analysis for longitudinal data. Adv. Psychol. Sci..

[65] Wens M.L., Mwangi M.N., van Loon A.F., Aerts J.C. (2021). Complexities of drought adaptive behaviour: Linking theory to data on smallholder farmer adaptation decisions. Int. J. Disaster Risk Reduct..

[66] Khan I., Lei H., Shah I.A., Ali I., Khan I., Muhammad I., Huo X., Javed T. (2020). Farm households’ risk perception, attitude and adaptation strategies in dealing with climate change: Promise and perils from rural Pakistan. Land Use Policy.

[67] Tesfaye A., Hansen J., Kassie G.T., Radeny M., Solomon D. (2019). Estimating the economic value of climate services for strengthening resilience of smallholder farmers to climate risks in Ethiopia: A choice experiment approach. Ecol. Econ..

[68] Musolino D.A., Massarutto A., Carli A. (2018). Does drought always cause economi losses in agriculture? An empirical investigation on the distributive effects of drought events in some areas of Southern Europe. Sci. Total Environ..

[69] Li W., Ruiz-Menjivar J., Zhang L., Zhang J. (2021). Climate change perceptions and the adoption of low-carbon agricultural technologies: Evidence from rice production systems in the Yangtze River Basin. Sci. Total Environ..

[70] Song Y., Zhang L., Zhang M. (2022). Research on the impact of public climate policy cognition on low-carbon travel based on SOR theory—Evidence from China. Energy.

[71] Berchin I.I., Nunes N.A., de Amorim W.S., Zimmer G.A.A., da Silva F.R., Fornasari V.H., Sima M., de Andrade J.B.S.O. (2019). The contributions of public policies for strengthening family farming and increasing food security: The case of Brazil. Land Use Policy.

